# Quality of healthcare services and its relationship with patient safety culture and nurse-physician professional communication

**DOI:** 10.15171/hpp.2017.30

**Published:** 2017-06-14

**Authors:** Akram Ghahramanian, Tayyebeh Rezaei, Farahnaz Abdullahzadeh, Zahra Sheikhalipour, Iman Dianat

**Affiliations:** ^1^Hematology and Oncology Research Center, Medical Surgical Department, Tabriz University of Medical Sciences, Tabriz, Iran; ^2^Student Research Committee, Department of Medical Surgical Nursing, Tabriz University of Medical Sciences, Tabriz, Iran; ^3^Department of Medical Surgical Nursing, Tabriz University of Medical Sciences, Tabriz, Iran; ^4^Department of Occupational Health and Ergonomics, Tabriz University of Medical Sciences, Tabriz, Iran

**Keywords:** Healthcare quality, Nurse-physician communication, Patient safety culture, Safety, Quality

## Abstract

**Background:** This study investigated quality of healthcare services from patients’ perspectives and its relationship with patient safety culture and nurse-physician professional communication.

**Methods: ** A cross-sectional study was conducted among 300 surgery patients and 101 nurses caring them in a public hospital in Tabriz–Iran. Data were collected using the service quality measurement scale (SERVQUAL), hospital survey on patient safety culture (HSOPSC) and nurse physician professional communication questionnaire.

**Results:** The highest and lowest mean (±SD) scores of the patients’ perception on the healthcare services quality belonged to the assurance 13.92 (±3.55) and empathy 6.78 (±1.88) domains,respectively. With regard to the patient safety culture, the mean percentage of positive answers ranged from 45.87% for "non-punitive response to errors" to 68.21% for "organizational continuous learning" domains. The highest and lowest mean (±SD) scores for the nurse physician professional communication were obtained for "cooperation" 3.44 (±0.35) and "non-participative decision-making" 2.84 (±0.34) domains, respectively. The "frequency of reported errors by healthcare professionals" (B=-4.20, 95% CI = -7.14 to -1.27, P<0.01) and "respect and sharing of information" (B=7.69, 95% CI=4.01 to 11.36, P<0.001) predicted the patients’perceptions of the quality of healthcare services.

**Conclusion: ** Organizational culture in dealing with medical error should be changed to non-punitive response. Change in safety culture towards reporting of errors, effective communication and teamwork between healthcare professionals are recommended.

## Introduction

Hospitals, as the most important element of the healthcare system, are aimed to provide a high quality care to patients and to meet their needs and expectations.^1^ Therefore, the institutionalization of quality in hospitals seems to be obligatory.^2^ Patients’ perceptions of the quality of healthcare services can influence the quality of healthcare services. In this respect, the availability of credible information about patients’ perceptions and expectations of the quality of healthcare services is required.^3^ According to the quality association of the United States, quality is defined as the ability for the manufacture of a product or provision of services so that customers’ needs are satisfactorily met.^4^ In addition, Headley and Bowen have also defined the quality of services as the difference between customers’ needs and what they really receive.^5^ The SERVQUAL model for measuring the quality of service was designed by Parasuraman et al.^6^ This model initially assessed the quality of services in ten domains. Then, the authors reduced the number of factors to five including reliability, responsiveness, assurance, empathy, and tangibles.^6^

Other investigators have recognized the quality of care as a multi-dimensional concept, in which patient safety is one of the most important and influential dimensions.^7^ Therefore, one of the basic goals of healthcare settings is the preservation, protection and improvement of patient safety.^7^ Patient safety as an essential component of quality of healthcare services is defined as the prevention of harm to the patient during the provision of healthcare services.^8^ The strong motivation for improving patient safety as a common international priority led to the world health organization’s resolution in 2002. According to this resolution, different countries were obliged to monitor and strengthen the safety of their healthcare systems,^9^ assess the culture of patient safety, remove weaknesses and develop safety culture among healthcare staff.^10^ It has been acknowledged that the improvement of the quality of healthcare services needs the improvement of communication within the organization and managers’ support, as well as positive attitudes toward patient safety.^11^

In the domain of internal communications, disrupted communication between nurses and physicians can also hinder teamwork and consequently endangers the safety and quality of care.^12^ Interactive communication between nurses and physicians is defined as the mutual engagement between them for the provision of care to patients and achievement of the common goal of healing.^13^ The difficulties of communication between physicians and other healthcare team members not only lead to practice errors and disruption of patient safety, but also lead to losing the patient’s trust, dissatisfaction and anger toward healthcare service providers.^12^

Attention to the society’s health promotion and the improvement of the patient’s satisfaction through the provision of high-quality healthcare services have been the main goals of the Iranian government in the health sector in the third and fourth development plans.^14^ The achievement of these goals requires different interventions including research in this area. The main prerequisite for success in achieving these goals is knowledge development and its application in practice.^15^ However, a review of the literature indicates that patients do not experience expected services from hospital and why this happens and what factors are influential that need to be investigated. Despite the importance of this issue, there is limited knowledge about the influencing factors such as the patient safety culture and nurse-physician professional communication on the quality of healthcare services. The aim of this study was to investigate quality of healthcare services from patients’ perspectives and its relationship with the patient safety culture and nurse-physician professional communication.

## Materials and Methods

### Study design, participants and procedure

This cross-sectional study was conducted in 2015 in the surgical wards of a public hospital in Tabriz, in the northwest of Iran. Data collection period was between October and December 2015. The study population consisted of all surgery patients older than 20 years in emergency and outpatient clinics. The sample size was determined based on an unpublished pilot study (by the current authors) on the correlation between patients’ perceptions of the quality of services and patient safety culture (r = 0.16) that based on this effect size, α = 0.05 and β = 0.80, the minimum sample size required using the two-tailed test via the G-Power software was calculated 266 patients. Considering the 10% probability of sample attrition, the number of participants was increased to 300, which were selected using a random sampling method. For this, a simple random sampling was applied using the Excel software to generate a list of random numbers from all patients who were on the surgery list before admission to surgery wards. Then, the first author selected numbers by the Excel software so that the sample size was obtained‏. In addition, a total of 101 nurses who cared for patients in the surgical wards of that hospital were also chosen using a census sampling method. Data was collected using questionnaires (from both nurses and their patients) as described in the following section. All study participants were familiarized with the study details and instruction was provided regarding the completing the questionnaires.

Participating nurses were asked to answer the questionnaires (after taking informed consent) and then mean scores of the safety culture and nurse-physician communication was calculated for each nurse. Staffing method in surgical wards (e.g. distribution of patients between nurses or case method) in each shift (morning, evening and night shifts) was also recorded in order to match the nurse responsible for each patient. Then, patients were interviewed in the discharge day regarding overall expectations and perceptions in relation to the quality of services received during a hospital stay based on service quality measurement scale (SERVQUAL) questionnaire. Finally, the relationship between quality of service (obtained from patients) and safety culture and nurse-physician professional communication (obtained from nurses caring patients) was investigated‏.

### Measures

The Persian version of the SERVQUAL was used to collect data regarding the patients’ perceptions of the quality of healthcare services.^16^ This scale was consisted of 32 items classified under two 15-question sets (with the 5-point Likert scale from very much = 5, much = 4, intermediate = 3, low = 2, very low = 1) for evaluation of expectations and perceptions. Two extra questions in this questionnaire were related to the overall quality of healthcare services. Questions 1-3 were related to “tangibles” domain. Questions 4-6, 7-9, and 10-13 were related to “reliability”, “responsiveness”, and “assurance” domains, respectively. Lastly, questions 14-15 were related to “empathy” domain. The psychometric properties of the Persian version of this questionnaire have been assessed and confirmed previously.^16^ The internal consistency for the total questionnaire was 0.97.^16^

The hospital survey on patient safety culture (HSOPSC) questionnaire was used to assess the patient safety culture in the hospital. This tool, designed by the Agency for Health Care Research Quality in the United States in 2004,^17^ consisted of 42 statements about staff’s perceptions of patient safety culture under the following domains: (1) “teamwork within units”, (2) “supervisor/manager expectations and actions promoting patient safety”, (3) “organizational learning–continuous”, (4) “management support for patient safety”, (5) “overall perceptions of patient safety”, (6) “feedback and communication about error”, (7) “communication openness”, (8) “frequency of events reported”, (9) “teamwork across units”, (10) “staffing”, (11) “handoffs and transitions” and (12) “non-punitive response to errors”. The response scale for domains 1-9 was based on a 5-point Likert scale (completely agree = 5, agree = 4, neutral = 3, disagree = 2, completely disagree = 1). The response scale for domains 10-12 was also based on a 5-point Likert scale (from always to never with a range of 5-1). There were also 2 additional questions regarding the consequences of developing patient safety culture (in addition to 42 items) including the frequency of reported errors during the last 12 months by healthcare professionals and the overall hospital score of patient safety. The validity and reliability of this questionnaire in Persian language has been confirmed previously.^18^ The means of positive answers were calculated for data analysis of this questionnaire. Based on the scoring system of this questionnaire, the domains with a mean percentage of positive answers of >70% were considered as the strength of patient safety culture, 50%-70% as neutral and <50% as the weakness of patient safety culture.

The nurse-physician professional communication questionnaire, designed by Rostami et al^19^ for nurses, was used in present study, which consisted of 22 items with 5 domains of “cooperation”, “respect and sharing of information”, “dictating perspectives and duties”, “non-participative decision-making” and “responding to errors and negligence”. Responses in this questionnaire were based on a 5-point Likert scale (completely agree = 5, agree = 4, neutral = 3, disagree = 2, and completely disagree = 1). The psychometric properties of this questionnaire have also been evaluated previously.^19^

The reliability analysis using the Cronbach’s alpha coefficient for the SERVQUAL, HSOPSC and the nurse-physician professional communication questionnaires was performed in a pilot study of 20, 10 and 10 subjects who were selected using convenient sampling method, respectively. The Cronbach’s alpha coefficients for the above-mentioned questionnaires and their domains were as: SERVQUAL = 0.78–0.95; HSOPSC = 0.70–0.75; and the nurse-physician professional communication questionnaire = 0.77–0.91. Once the reliability of the three measures had been established, permission was obtained to access the research sites in the hospital, and then data were collected by the investigators. The nurses’ and patients’ questionnaires took approximately 20-25 minutes to complete.

### Statistical analyses

The collected data were analyzed using descriptive and inferential statistics via the SPSS software version 22 (IBM Inc., Armonk, NY, USA). Means (percentages) and frequencies were reported for quantitative and qualitative variables, respectively. Pearson correlations coefficients and multiple linear regression analysis were used to determine the relationships between study variables and predictive factors influencing the patients’ perceptions of the quality of healthcare services. It should be noted that patient safety culture and nurse-physicians professional communication were entered as independent variables and the patients’ perceptions of the quality of healthcare services was entered as depended variable in the regression model (step- by step) for complementary data analysis.* P* values less than 0.05 were considered as statistically significant.

## Results

Patients had a mean age of44.99, mean hospital stay of 1.31 days, and the of them were male (57.7%), married (78.3%) and candidate for ENT surgery (27.7%) (Table 1). Participating nurses had a mean age of37.06. Their mean working experience in nursing and surgery ward were12.43 years and 6.78 years, respectively. Their mean weekly working hours was 44.77, and the majority of them were female (85.1%) and married (82.2%) (Table 2).

The mean (± SD) of SERVQUAL scores for expectation and perception of service were 66.59 (±8.52) and 51.81 (±11.97) respectively (Table 3). The highest mean score of the patients’ perception regarding the quality of healthcare services belonged to the assurance domain with 13.92 (± 3.55), while the least mean score was related to the empathy domain with 6.78 (±1.88). The results showed a gap between the patients’ perceptions of the quality of healthcare services (service) and real conditions in the surgical wards (expectation of service) (mean ± SD = -14.78 ± 13.63).

With regard to the patient safety culture, the mean percentage of positive answers of the HSOPSC was 59.85 and ranged from 45.89% (non-punitive response to errors) to 68.21% (organizational continuous learning). The mean percentage of positive responses observed in this present study show no strength in none of the domains of the patient safety culture. The non-punitive response to errors, together with staffing, was identified as the weakness of the patient safety culture, while the other domains and total score of patient safety culture were considered as neutral (Table 4).

With regard to the nurse-physician professional communication, the mean (± SD) of total score was 3.11 (± 0.24) and mean scores for the different domains (in order of decreasing score) were as: cooperation = 3.44 (± 0.35); responding to errors and negligence = 3.29 ± (0.15); dictating perspectives and duties = 3.01 (± 0.23); respect and sharing of information = 2.99 (± 0.35); and non-participative decision-making = 2.84 (± 0.34).

The results of the Pearson correlation coefficients between the study variables are presented in Table 5. A number of significant correlations were found between the study variables including that of the patients’ perceptions of the quality of healthcare services with patient safety culture (*r* = 0.82; *P* < 0.001) and nurse-physician professional communication (*r* = 0.16; *P* < 0.01).

The results of the multivariate analysis shown that “frequency of reported errors by healthcare professionals” (B = -4.20, 95% CI = -7.14-1.27, *P* < 0.01) and “respect and sharing of information” (B = 7.69, 95% CI = 4.01-11.36, *P* < 0.001) predicted the patients’ perceptions of the quality of healthcare services. This means that for each 1 score increase in “the respect and sharing of information between nurses and physicians”, the patients’ perceptions of the quality of healthcare services increased by 7.69 times. Moreover, 1 score increase in “the frequency of reported errors”, decreased the patients’ perceptions of the quality of healthcare services by 4.20 times (Table 6).

## Discussion

The aim of this study was to investigate quality of healthcare services from patients’ perspectives and its relationship with the patient safety culture and nurse-physician professional communication in surgical units of a hospital in Tabriz, Iran. One of the main findings was that the highest and lowest mean scores of the patients’ perceptions of the quality of healthcare services belonged to the assurance and empathy, respectively. The results of an Iranian study on the quality of healthcare services showed that the highest mean scores of patients’ perceptions were related to the assurance and reliability domains,^20^ which is, in part, similar to our results. In addition, the results of another study conducted in hospitals affiliated with the social security organization in Tehran showed that the highest and lowest mean score of perceived quality belonged to the reliability and responsiveness, respectively.^21^ In the other hand, the findings of another study conducted in Zanjan, Iran, indicated that the highest mean score of perceived quality was related to the tangibles domain and the lowest score was obtained for the reliability domain.^22^ And finally, the findings of a study in Zahedan, Iran showed that the highest and lowest mean scores of the patients’ perceptions were related to tangibles and empathy domains, respectively.^23^ These findings may reflect the varied patterns of healthcare services delivered in different areas. However, according to our findings, the healthcare services were delivered in an accurate, on time and reliable manner, although the presence of low levels of responsiveness of healthcare staff, on time performance of tasks, informing the patients about the time of providing care, psychological support of and paying attention to the patients’ needs were considered as shortcomings in this regard. Therefore, these shortcomings call for a reform in the provision of care with the consideration of empathic care and responsiveness toward the patients’ needs, which can consequently lead to improved health promotion and quality of services.

As shown in this research, there was a negative gap (gap mean = -14.78) between the patients’ expectations and perceptions of the quality of healthcare services so that their expectations were beyond their perceptions. This finding is in line with several previous researches in Iran^24,25^ and elsewhere.^26,27^ (Results of previous research should be mentioned in the introduction.) In the other hand, there are also some studies in other countries such as Spain^28^ and Malaysia,^29^ which have shown a positive gap between the patients’ expectations and perceptions of the quality of healthcare services. Such a difference may be attributed to the difference between our study setting which was a public hospital and private hospitals evaluated in other studies. It is believed that with less nursing shortages and patients’ overcrowding in private hospitals, the quality of healthcare services is high.

With regard to the patient safety culture, the highest mean scores in our study were related to the organizational learning and management support for patient safety domains, while the lowest scores were obtained for the non-punitive responses to errors and staff working issues. This finding suggests the need to establish an environment that encourages the reporting of errors. This finding is relatively similar to the findings of some previous investigations in Iran^30,31^ and other countries.^32^ The finding that the lowest score belonged to the non-punitive responses to errors and staff working issues, together with the recommendation of the American Medical Association,^33^ provides additional evidence that healthcare organizations need to seize the opportunity of learning from mistakes due to the failure of individuals.^34^ The results of the present study also indicated a negative relationship between the frequency of reported errors and the patients’ perceptions of the quality of healthcare services. Other investigators have acknowledged that the non-punitive responses to errors can be regarded as an important indicator of the patient safety culture as it has the potential to improve the reporting of errors, and consequently help to root cause analysis of errors and devising strategies to prevent future error incidents.^35^

The results of the present study suggest that the nurse-physician professional communication has a potential to improve the patients’ perceptions of the quality of healthcare services. It has been reported that the nurse-physician communication could lead to the improvement of their collaboration and the quality of patient care.^36^ It has also been acknowledged that the nurses’ collaboration in decision-making can improve their feeling of responsiveness and performance, positive attitudes toward the organization, job satisfaction and quality of care.^37^ Additionally, the results of the present study showed that the nurse-physician professional communication and patient safety culture were positively related to the perceptions of the quality of healthcare. Gillespie et al in an ethnographical study reported that a gap in the communication between surgery healthcare team members could lead to patient harm, and therefore the authors suggested that the development of a patient safety culture in the hospital should be established based on open discussions on teamwork and mutual expectations between healthcare staff.^38^ Taken together, these findings provide further evidence that the creation of an atmosphere of mutual respect between physicians and nurses and sharing information between them, instead of the tradition of physician-domination, can improve the quality of healthcare services, and consequently patient outcomes.

### Study limitations and future work

The results of this study are related to the surgical wards of a hospital in Tabriz, Iran (Imam Reza hospital) and may not be generalizable to other hospitals or clinical settings in Iran or elsewhere. Therefore, further studies in other settings may be required to fully justify our findings. Moreover, as the findings of this study showed a gap between the patients’ perceptions of the quality of healthcare services and real conditions in the surgical wards, this may be a basis for further studies in future.

## Conclusion

In conclusion, the results of this study highlight the need for improvement of perceived quality of healthcare services (especially empathy with patients) as well as the weaknesses of patient safety culture (and in particular non-punitive response to errors) and nurse-physician professional communication skills (particularly respect and sharing of information and non-participative decision-making) in healthcare settings. The results also suggest the need for designing strategies such as the change in hospital culture towards reporting of errors and effective communication and teamwork between healthcare professionals, which can consequently influence the quality of healthcare services and patient outcomes.

## Ethical approval

The study protocol was reviewed and approved by the ethics committee of the Tabriz University of Medical Sciences (Ethics code: TBZMED.REC.1394.525). Informed consent was obtained from all study participants prior to data collection.

## Competing interests

The authors declare that there is no conflict of interests.

## Disclaimer

The authors claim that no part of this manuscript has been copied from other sources.

## Authors’ contributions

AG involved in the conceptualization of the study. AG and FA involved in the analysis and interpreting the data for the work. TR and ZS involved in the designing of the work and data collection. AG and TR involved in the drafting the manuscript. ID involved in the data interpretation and editing the work.

## Funding

This study was financially supported by the vice chancellor of research in Tabriz University of Medical sciences (Grant No: 540).

## Acknowledgments

The authors greatly appreciate all the hospital authorities as well as all head nurses, nurses and patients who collaborated in this study.

**Table 1:** Demographic characteristics of patients (n = 300)

**Variable**	**No.**	**%**
Surgery type		
General	57	19
ENT (ear, nose and throat)	83	27.7
Urology	50	16.7
Neurology	37	12.3
Jaw and Face	13	4.3
Thorax	35	11.7
Orthopaedic	5	1.7
Kidney transplant	7	2.3
Trauma	13	4.3
Gender		
Male	173	57.7
Female	127	42.3
Income level		
Low	10	3.4
Moderate	124	42.0
High	161	54.6
Marital status		
Single	65	11.7
Married	235	78.3
Educational level		
Illiterate	96	32.0
Primary	70	23.3
Secondary	99	33.0
University	35	11.7
Insurance type		
Social security insurance	106	35.0
Health service	21	7.0
Rural insurance	64	21.3
Armed forces insurance	19	6.3
Health insurance	69	23.0
Others	21	7.0
Age		
Mean (SD) = 44.99 (17.53)	–	–
Hospitalized days		
Mean (SD) = 2.91 (2.75)	–	–
Duration of hospitalization (day)		
Mean (SD) = 1.31 (0.68)	–	–

**Table 2:** Demographic and job details of nurses (n = 101)

**Variable**	**No.**	**%**
Wards		
General	21	20.8
ENT (ear, nose and throat)	8	7.9
Urology	14	13.9
Neurology	9	8.9
Jaw and Face	6	5.9
Thorax	11	10.9
Orthopaedic	10	9.9
Kidney transplant	9	8.9
Trauma	13	12.9
Gender		
Male	15	14.9
Female	86	85.1
Employment status		
Full time	32	31.7
Part time	69	68.3
Marital status		
Single	18	17/87
Married	83	82/2
Educational level		
Undergraduate	92	91/1
Postgraduate	9	8/9
Age (years)		
Mean (SD) = 37.06 (2.03)	–	–
Total job experience (years)		
Mean (SD) = 12.73 (2.15)	–	–
Job experience in the current profession (years)		
Mean (SD) = 12.43 (2.10)	–	–
Job experience in the relevant unit (years)		
Mean (SD) = 6.78 (2.36)	–	–
Weekly working hours		
Mean (SD) = 44.77 (2.39)	–	–

**Table 3:** Mean (SD) of SERVQUAL scores

**Dimensions**	**Expectation of service**		**Service perception**		**Service gap** ^a^	
	**Mean**	**SD**	**Mean**	**SD**	**Mean**	**SD**
Tangibles (3 items)	13.17	1.84	10.32	2.61	-2.84	2.91
Reliability (3 items)	13.26	1.79	10.65	2.40	-2.57	2.70
Responsibility (3 items)	13.32	1.86	10.13	2.71	-3.16	3.14
Assurance (4 items)	17.93	2.48	13.92	3.55	-3.98	4.13
Empathy (2 items)	8.90	1.28	6.78	1.88	-2.10	2.15
Total score	66.59	8.52	51.81	11.97	-14.78	13.63

The difference between service perception and expectation of service.

**Table 4:** Mean percentage of positive answers of the HSOPSC

**Dimensions**	**Mean % of positive answers**	**Judgment**
Teamwork within units	65.90	Neutral
Supervisor/manager expectations & actions promoting patient safety	63.25	Neutral
Organizational learning—continuous improvement	68.21	Neutral
Management support for patient safety	66.47	Neutral
Overall perceptions of patient safety	66.57	Neutral
Feedback & communication about error	63.28	Neutral
Communication openness	58.75	Neutral
Frequency of events reported	56.42	Neutral
Teamwork across units	54.98	Neutral
Staffing	45.89	Weakness
Handoffs & transitions	62.63	Neutral
Non-punitive response to errors	45.87	Weakness
Total score	59.85	Neutral

**Table 5:** Correlation matrix between quality of healthcare services and other variables

**Variables**	**2**	**3**	**4**	**5**	**6**	**7**	**8**	**9**
1. Expectation of service	0.14^*^	0.69	-0.02	-0.03	0.04	0.01	0.04	0.01
2. Service perception		0.16^*^	0.82^**^	-0.11	-0.02	0.19^**^	0.20^**^	0.22^**^
3. Nurse-physician communication			0.09	-0.06	0.1	0.56^**^	0.47^**^	0.78^**^
4. Safety culture				-0.01	0.18^*^	0.66^**^	0.50^**^	0.66^**^
5. Patient age					-0.001	-0.04	-0.06	-0.07
6. Hospitalization day						0.06	-0.01	0.08
7. Hospital stay							-0.03	-0.01
8. Nurses’ age								0.88^**^
9. Nurses’ experience in nursing								

* *P* < 0.01, ** *P* < 0.001.

**Table 6:** Regression analysis of variables related to quality perception of received services

**Variables**	**B (95% CI)**	**Beta**	***P*** ** value**
Respect and sharing of information	7.69 (4.01-11.36)	0.23	<0.001
Frequency of reported errors	-4.20 (-7.14--1.27)	-0.15	<0.01
